# Assessing Autophagy in Archived Tissue or How to Capture Autophagic Flux from a Tissue Snapshot

**DOI:** 10.3390/biology9030059

**Published:** 2020-03-21

**Authors:** Magali Humbert, María Morán, Patricia de la Cruz-Ojeda, Jordi Muntané, Tabea Wiedmer, Nadezda Apostolova, Sharon L. McKenna, Guillermo Velasco, Walter Balduini, Leopold Eckhart, Bassam Janji, Belém Sampaio-Marques, Paula Ludovico, Eva Žerovnik, Rupert Langer, Aurel Perren, Nikolai Engedal, Mario P. Tschan

**Affiliations:** 1 TRANSAUTOPHAGY: European Network for Multidisciplinary Research and Translation of Autophagy Knowledge, COST Action CA15138, 08193 Barcelona, Spain; mmoran@h12o.es (M.M.); jmuntane-ibis@us.es (J.M.); nadezda.apostolova@uv.es (N.A.); s.mckenna@ucc.ie (S.L.M.); gvelasco@quim.ucm.es (G.V.); walter.balduini@uniurb.it (W.B.); leopold.eckhart@meduniwien.ac.at (L.E.); bassam.janji@lih.lu (B.J.); mbmarques@med.uminho.pt (B.S.-M.); pludovico@med.uminho.pt (P.L.); eva.zerovnik@ijs.si (E.Ž.); Nikolai.Engedal@rr-research.no (N.E.); 2 Institute of Pathology, University of Bern, Murtenstrasse 31, CH-3008 Bern, Switzerland; TWiedmer@cemm.oeaw.ac.at (T.W.); rupert.langer@pathology.unibe.ch (R.L.); aurel.perren@pathology.unibe.ch (A.P.); 3 Mitochondrial and Neuromuscular Diseases Laboratory, Instituto de Investigación Sanitaria Hospital ‘12 de Octubre’ (‘imas12’), 28041 Madrid, Spain; 4 Spanish Network for Biomedical Research in Rare Diseases (CIBERER), U723, Institute of Health Carlos III (ISCIII), 28029 Madrid, Spain; 5 Institute of Biomedicine of Seville (IBiS), Hospital University “Virgen del Rocío”/CSIC/University of Seville, 41013 Seville, Spain; patricia.cruz.ojeda@gmail.com; 6 Department of Surgery, School of Medicine, University of Seville, 41009 Seville, Spain; 7 Spanish Network for Biomedical Research in Hepatic and Digestive Diseases (CIBERehd), Institute of Health Carlos III (ISCIII), 28029 Madrid, Spain; 8 Department of Pharmacology, University of Valencia, 46010 Valencia, Spain; 9 Cancer Research at UCC, Western Gateway Building, University College Cork, T12 XF62 Cork, Ireland; 10 Department of Biochemistry and Molecular Biology, School of Biology, Complutense University, and Instituto de Investigaciones Sanitarias San Carlos (IdISSC), 28040 Madrid, Spain; 11 Department of Biomolecular Sciences, University of Urbino Carlo Bo, 61029 Urbino, Italy; 12 Department of Dermatology, Medical University of Vienna, Vienna 1090, Austria; 13 Tumor Immunotherapy and Microenvironment (TIME) Group, Department of Oncology—Luxembourg Institute of Health, 1526 Luxembourg City, Luxembourg; 14 Life and Health Sciences Research Institute (ICVS), School of Medicine, University of Minho, 4710-057 Braga, Portugal; 15 ICVS/3B’s—PT Government Associate Laboratory, Braga/Guimarães, Portugal; 16 Department of Biochemistry and Molecular and Structural Biology, Jožef Stefan Institute, 1000 Ljubljana, Slovenia; 17 Department of Tumor Biology, Institute for Cancer Research, Oslo University Hospital, 0424 Oslo, Norway

**Keywords:** autophagy, biomarkers, pathology, disease

## Abstract

Autophagy is a highly conserved degradation mechanism that is essential for maintaining cellular homeostasis. In human disease, autophagy pathways are frequently deregulated and there is immense interest in targeting autophagy for therapeutic approaches. Accordingly, there is a need to determine autophagic activity in human tissues, an endeavor that is hampered by the fact that autophagy is characterized by the flux of substrates whereas histology informs only about amounts and localization of substrates and regulators at a single timepoint. Despite this challenging task, considerable progress in establishing markers of autophagy has been made in recent years. The importance of establishing clear-cut autophagy markers that can be used for tissue analysis cannot be underestimated. In this review, we attempt to summarize known techniques to quantify autophagy in human tissue and their drawbacks. Furthermore, we provide some recommendations that should be taken into consideration to improve the reliability and the interpretation of autophagy biomarkers in human tissue samples.

## 1. Autophagy at Glance

**Figure 1:**
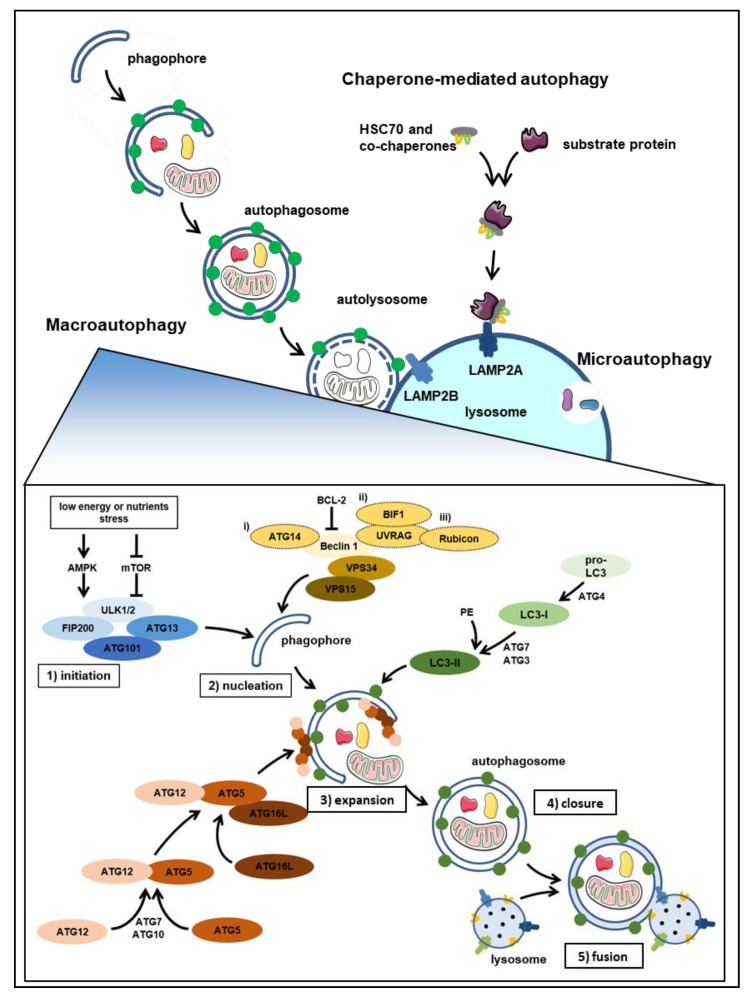
The autophagy machinery: Macroautophagy: After initiation, several autophagy proteins, including LC3B and the ATG12-ATG5-ATG16L1 complex are recruited to the pre-autophagosomal site. Next, the phagophore is expanded and in parallel cytoplasmic content gets engulfed before the autophagosome closes and finally fuses with the lysosome. Chaperone-mediated autophagy (CMA): The molecular chaperone HSC70 binds potential substrates containing the KFERQ (Lys-Phe-Glu-Arg-Gln) amino acid motif in the cytosol and targets them to the lysosomal surface via the LAMP2A receptor on the lysosome membrane, leading to the degradation of the substrate by lysosomal proteases. Microautophagy; the lysosomal membrane undergoes invaginations and/or protrusions to directly internalize and degrade cytoplasmic material.

Autophagy is a dynamic process that controls cellular homeostasis, stress adaptation, and under certain conditions, regulates cell death in eukaryotes [1,2]. Deregulation of autophagy is found in a variety of human pathologies. Autophagy is, therefore, attracting an increasing number of scientists who investigate this degradation mechanism in cell culture systems, animal models, and patient samples. Three main autophagy pathways have been described: macroautophagy, chaperone-mediated autophagy (CMA), and microautophagy (Figure 1).

Macroautophagy is characterized by the formation of double-membraned vesicles, called autophagosomes that engulf superfluous or harmful components of the cytoplasm. Autophagosomes are directed towards lysosomes, where they fuse to degrade and recycle their contents [3]. Macroautophagy is a multistep process divided into initiation, nucleation, expansion, closure, and fusion. These steps are regulated by six autophagy-related (ATG) [4] protein classes/complexes [5,6,7,8,9,10]; (i) The unc-51-like kinase 1 (ULK1) complex, composed of ULK1, FIP200, ATG13 and ATG101; ATG9; (ii) the class III PI3K complex, composed of VPS34, Beclin1 and vesicular transport factor p115; (iii) WIPI proteins; and (iv and v) the two ubiquitin (ub)-like conjugation systems, the ub-like ATG12 conjugates, composed of ATG12 and ATG5 and the ub-like ATG8 (LC3 and GABARAP proteins), which conjugates to phosphatidylethanolamine and (iv) the less studied ATG2-ATG18 complex. The conjugation systems critically involve ATG3, ATG7, ATG10, and ATG16L1, whereas ATG4 is required for ATG8 activation. Several signals have been shown to activate the **initiation** of the macroautophagy process. All of them target the ULK1 complex, which in turn triggers **nucleation** of the phagophore by phosphorylating components of the class III PI3K complex. The class III PI3K complex stimulates local phosphatidylinositol-3-phosphate (PI3P) production allowing the recruitment of WIPIs (and other proteins) to the membrane. WIPIs attract the ATG12 complex (ATG12-ATG5-ATG16L1) to the phagophore leading to lipid conjugation of ATG8 proteins (in conjunction with the activity of ATG7 and ATG3). The activation of these two conjugation systems leads to expansion of the double membrane. Recently, reports demonstrated that lipid transfer of ATG9A vesicle delivery necessary for the phagophore expansion is dependent on ATG2A/B ATG9 interaction at the mitochondria-associated endoplasmic reticulum (ER) membrane [11,12,13]. In addition, mechanistic studies propose that ATG2A/B is responsible for the expansion and closure of the phagophore via binding to GABARAP/GABARAP-L1 independently of WIPI4 [14]. Once **closed,** the double-membraned vesicle undergoes intracellular trafficking and may fuse with endosomes (forming an amphisome) and/or fuse directly with the lysosome. In addition, the macroautophagic pathway is tightly linked to endocytosis. The endocytic pathway facilitates the nucleation and maturation of the phagophore and the lysosome-autophagosome fusion (reviewed in [15,16]). In line, inhibiting endocytosis using Dynasore leads to autophagy inhibition, despite the fact that mTORC1 activity is decreased [17], reinforcing the positive interplay between endocytosis and autophagy.

Chaperone-mediated autophagy (CMA) is a selective autophagy pathway that modulates the turnover of soluble cytosolic proteins. In contrast to macroautophagy, CMA delivers cargo directly to the lysosomes. Therefore, CMA does not require the formation of vesicles [18,19,20]. CMA is a multistep process that is initiated by (1) recognition of the substrate (2) substrate binding and unfolding followed by (3) the translocation of substrate to the lysosome and finally (4) degradation of the substrate within the lysosome [19]. Briefly, the molecular chaperone HSC70 (HSPA8) recognizes and binds CMA substrates containing a KFERQ (Lys-Phe-Glu-Arg-Gln)-like amino acid motif [21] within the cytosol and targets them to the lysosomal surface. At the lysosome, the substrate interacts with the cytosolic tail of the lysosome-associated membrane protein (LAMP) type 2A receptor. This interaction leads to the multimerization of LAMP2A at the lysosomal membrane, stabilized by HSP90, which is located on the luminal side of the lysosome membrane. Substrates are finally translocated and degraded by lysosomal proteases.

Microautophagy is characterized by the degradation of intracellular proteins and organelles directly engulfed by lysosomes or endosomes. In mammalian cells three types of microautophagy exist: (a) Microautophagy with lysosomal protrusion, (b) Microautophagy with lysosomal invagination, and (c) Microautophagy with endosomal invagination. The latter, termed endosomal microautophagy (eµA), occurs in late endosomes/multivesicular bodies, which subsequently fuse with the lysosome for full degradation [22]. The detailed steps and molecular mechanisms of microautophagy are yet to be fully determined. Endosomal microautophagy has been described as a stepwise pathway, starting with an invagination of the membrane that is coordinated by ESCRT and accessory proteins. Similarly, to CMA, proteins bearing the KFERQ-like motif will be targeted via the binding of HSC70 (HSPA8) and then internalized. Various types of organellar microautophagy have been described in yeast [23,24]. The extent of organellar microautophagy in mammalian cells is yet to be determined.While the predominant role of autophagy is to promote cell survival, in specific contexts autophagy, the autophagic machinery, or components thereof can be either (i) associated with, (ii) contributing to, (iii) required for, or (iv) mediating regulated cell death (reviewed in [25,26]). The autophagic machinery or components thereof may be engaged or upregulated as part of stress responses alongside cell death in a futile attempt to restore homeostasis. However, there are also many examples of the involvement of ATGs or other autophagy-associated gene products in regulated cell death. For instance, ATG4D, ATG5, Beclin-1, MAP1LC3B, and p62/SQSTM1 have been implicated in apoptosis and caspase activation under various conditions [27,28,29,30,31,32]. Macroautophagic responses or components of the macroautophagic machinery can also contribute to cell death induction independently of apoptosis. For instance, in apoptosis-deficient *Bax/Bak* knockout mouse fibroblasts, chemotherapy-induced cell death requires ATG5 and Beclin-1 [33]. Moreover, autophagy can promote ferroptosis via degradation of ferritin [34]. Furthermore, necrosome assembly can occur on the autophagosomal membranes [35]. The caspase-independent regulated necrosis called necroptosis can be induced in a pan-caspase inhibited environment by TRAIL and TNF. TRAIL-induced necroptosis is ATG5 dependent while TNF-dependent necroptosis is ATG5 and ATG16L1 dependent [36]. Together, these links indicate that analyzes of autophagy and autophagic markers in tissues can provide information not only about autophagic activity, but also on various modes of cell death. Further studies on the roles of the different ATG proteins and other autophagy-associated proteins on cell death will improve the understanding of the relationship between autophagy and cell death.

## 2. Autophagy and Human Disease

Autophagy is an attractive research subject for the biomedical community because of its crucial role in maintaining organelle homeostasis, proteostasis, and the cellular energetic balance. Indeed, autophagy deregulation has been linked to many human disorders including neurodegenerative conditions, metabolic disorders, myopathies, heart conditions, and cancer. Therefore, efforts have been made to understand the function of autophagy in diseases to improve current therapies. Macroautophagy is modulated by a large number of clinical drugs, which affect various steps in the autophagic pathway, and include both inducers of autophagy (rapamycin/rapalogs, metformin, lithium, chlorpromazine, and others) and inhibitors (hydroxychloroquine, azithromycin, clomipramine, and others) (reviewed in [37]). In this section, we will summarize recently published insights into the roles of autophagy in a selected set of human diseases. For more detailed information, please see the indicated list of recent review articles [38,39,40,41,42,43,44,45,46,47].

### 2.1. Autophagy in Cancer

A tumor-suppressive function of macroautophagy is supported by animal models, for example Beclin1 heterozygous mice display an increased tumor incidence [48]. Conversely, stress- and cancer therapy-related induction of macroautophagy frequently supports tumor cell survival, suggesting an oncogenic function ([49,50]). Altogether, the role of macroautophagy in tumorigenesis is still controversial and likely depends on the type of tumor and the stage of disease progression [51] (Table 1).

Similar to macroautophagy, accumulating evidence suggests that CMA activity has a dual effect on tumor development and growth (Reviewed in [38]). High LAMP2A expression has been found in numerous cancer types and was linked to higher CMA activity [52]. Therefore, increased effort has been made to define the role of CMA in cancer. CMA may sustain the Warburg effect in cancer cells [53] either indirectly by targeting p53 and thus reducing p53-dependent transcription of key glycolytic enzymes such as GAPDH and aldolase [52], or directly by targeting key glycolytic enzymes such as acetylated PKM2 [54] and HK2 [55]. Degradation of PKM2 and HK2 leads to an accumulation of glycolytic intermediates and proliferative signals [53]. CMA may also improve resistance to various stress inducing stimuli including chemotherapy [56], ER stress [57] and hypoxia [58,59]. Lastly, CMA may contribute to tumor cell proliferation and metastatic potential [52,60,61,62]. While constitutively active CMA was frequently found in cancer cells [52], a study reported that the lack of Lamp2A expression in mice increased the risk of malignant transformation and liver tumorigenesis [63]. Further studies are warranted to better understand the impact of CMA on cellular transformation and on cancer cells.

#### 2.1.1. Solid Tumors

The different roles of autophagy in cancer seem to be related to the tumor type, stage, and genetic context [49]. Autophagy is likely to play as predominantly tumor suppressor role during the initiation and development of tumors. However, in well-established tumors, autophagy may be a survival mechanism in response to stress [64]. In accordance with a tumor suppressor role, a decreased expression of Beclin-1 at mRNA and protein levels have been found in human brain tumor samples, compared to non-tumoral lesions [65]. In the context of breast cancer, reduced *BECN1* mRNA expression contributes to poor prognosis in HER2-enriched breast tumors [66]. In addition, upstream positive regulators of Beclin-1, such as UV radiation, resistance-associated gene or Bax interacting factor-1 (Bif-1), have been found downregulated in several types of cancers, including colorectal cancer [67,68]. In contrast, Ras-driven tumors seem to be autophagy-dependent [69]. For instance, Ras-driven tumorigenesis in pancreatic or lung cancer likely relies on autophagy induction through oncogenic Ras pathway activation to promote cell transformation, reactive oxygen species (ROS) clearance and mitochondrial oxidative phosphorylation [70,71,72]. Correlative evidence suggests that resistance to systemic therapies based on tyrosine kinase inhibitors (TKIs) could be regulated by autophagy. In hepatocarcinoma (HCC), Sorafenib resistance was reported to be related to AMP-activated protein kinase (AMPK), which induces pro-survival autophagy and reduces cell death [73]. Upregulation of GATA6, a transcription factor that mediates the expression of autophagy-related genes such as ATG5, ATG7, and ATG12 by erlotinib treatment promotes treatment resistance in cellular models of non-small cell lung cancer (NSCLC) [74].

All this knowledge suggests that autophagy could serve as a targetable pathway to treat cancer progression, although controversies remain regarding whether to inhibit or enhance autophagy. Strategies based on the blockage of autophagy usually combine traditional inhibitors with cancer therapies. As an example, the use of chloroquine (CQ) during Sorafenib treatment in a thyroid cancer subcutaneous mice model, markedly reduced tumor volume and enhanced caspase-3 activation [75]. In HCC, combination of the PARP inhibitor Niraparib and CQ increased cell death and DNA damage [76]. Converesly, in other models of cancer, boosting autophagy has an anti-tumoral effect. Mitochondrial targeted Lonidamine (mito-LND) blocks mitochondrial bioenergetics, leading to ROS and autophagic-induced cell death, therefore, alleviating lung cancer progression [77]. Another strategy based on glycolytic metabolism inhibition in breast cancer promotes autophagy to bypass Lapatinib resistance [78].

Increasing evidence suggests that alterations in autophagy may be a major mechanism of tumor escape from immune surveillance by regulating signaling pathways in both tumor and immune cells [42]. Targeting autophagy in cancer cells in combination with other therapeutic strategies, such as immunotherapy, has gained significant interest to promote tumor regression. In fact, some authors have proposed autophagy-associated cell death as a key immunogenic mechanism that could potentiate tumor treatment response and mitigate progression [79,80,81]. In immune-competent animal models, autophagy is necessary for dendritic cell and T-lymphocyte infiltration [82,83,84]. Autophagic vesicles charged with defective ribosomal products (DRiPs) may serve as tumor vaccines to facilitate antigen cross-presentation and immunogenicity [85]. Dribble DRiPs based vaccines have been tested in clinical trials along with cyclophosphamide in patients with NSCLC, showing increased T and B-cell response, thus, serving as potential immunotherapy based on autophagy promotion (NTC01909752) [85].

#### 2.1.2. Leukemia and Lymphomas

Hematopoiesis is a highly hierarchical process during which hematopoietic cells produce functional progenitor cells including mature myeloid cells and lymphocytes. Deregulation of this process can lead to a block in differentiation of immature cells that have acquired increased self-renewal potential. One of the key mechanisms that maintains hematopoietic homeostasis is autophagy (reviewed in [41,45]. There is solid evidence that hematopoietic stem cells (HSCs) with impaired macroautophagy are more prone to ageing and have an increased risk of developing hematopoietic malignancies [86,87,88,89,90,91]. Lymphomas and leukemias are highly heterogeneous blood cancers [92,93]. Autophagy can act either as pro- or antiproliferative mechanism depending on the lineage and the genotype of the disease [41]. The complexity of the interplay between autophagy and disease progression in blood cancer is well exemplified in acute myeloid leukemia (AML) studies. Autophagy deregulation has been described in AML where ATG gene expression is frequently repressed [90,94,95,96,97]. Autophagy abrogation, by deletion of key ATG genes, leads to leukemia initiation and progression in mouse models [96,97,98,99]. Remarkably, some studies showed a high frequency of AML patients, particularly those with complex karyotypes, carrying heterozygous deletions, missense mutations or copy number variations of ATG genes [97,100,101]. Furthermore, a correlation was found between AML and heterozygous chromosomal loss of 5q, 16q, or 17p with the encoded regions for the ATG genes *ATG10* and *ATG12*, *GABARAPL2* and *MAP1LC3B*, or *GABARAP*, respectively [97]. AML is a highly heterogeneous disease, therefore it is expected that macroautophagy can be both tumor-promoting or suppressive. For instance, the pro-oncogenic FLT3-ITD mutation found in many AML patients promotes higher macroautophagy levels, suggesting that macroautophagy is not tumor-suppressive in this setting and may instead be tumor-promoting [102]. On the other hand, combining expression of the oncofusion protein MLL-ENL expression with knock out of *Atg7* or *Atg5* leads to a more aggressive leukemia in a mouse model, indicating a tumor-suppressive role of autophagy under those conditions [97]. Interestingly, using an MLL-Af9 leukemia model, Liu et al. reported that while macroautophagic activity is key for the disease development, it is dispensable for the maintenance of leukemia [103]. Therefore, although autophagy is critical in the maintenance of hematopoietic stem cells (HSCs), it plays context-dependent roles in leukemia initiation and progression, suggesting a highly complex role for autophagy in leukemic transformation and leukemic stem cells properties in AML (reviewed in [104]). To further complicate this scenario, autophagy is also established as one of the resistance mechanisms of leukemic cells to chemotherapy [94,95]. There are clinical trials in which hydroxychloroquine (an autophagy inhibitor), increased cytotoxicity of conventional chemotherapy in leukemic cells [98,105]. However, there is still divergence on this subject and it is accepted that autophagy has a versatile role, which will depend on the progenitor and the driver engaged in the leukemia transformation and the state of leukemic expansion. Of note, some oncofusion proteins, such as PML-RARA [106,107] and BCR-ABL [108,109], which are encoded in APL and CML, are degraded by autophagy, whereas others, such as AML1-ETO, are not [110].

### 2.2. Autophagy in other Diseases (Neurodegenerative Conditions, Metabolic Disorders, Myopathies, and Heart Conditions)

In addition to influencing the initiation and progression of cancer, deregulation of autophagy has been linked to other human diseases such as neurodegenerative conditions, metabolic disorders, and autoimmune diseases.

#### 2.2.1. Neurodegenerative Disorders

Neurodegenerative diseases are characterized by aggregation of misfolded proteins and loss of neuronal population [119]. Many mutated proteins in neurodegenerative diseases are autophagy targets (e.g., mutated α-synuclein in Parkinson’s disease, mutated huntingtin in Huntington’s disease or mutants of TAR DNA-binding protein 43 (TDP-43) in amyotrophic lateral sclerosis) [120]. Loss of key ATG genes such as *Atg5* or *Atg7*, has been shown to accelerate neurodegeneration in mouse models [121,122]. Therefore, activating autophagy in neurodegenerative disorders may be beneficial [123]. In concordance, mice with a conditional deletion of *Atg7* in dopamine neurons showed progressive neuron loss accompanied by p62-positive inclusions [124]. Surprisingly, elderly mice had increased dopamine neurotransmission leading to improved movement [124]. Those results indicate that although macroautophagy may be beneficial for neuronal survival, impairing autophagy in Parkinson’s disease patients could improve their motor performance. It is important to note that ATG7, like most of the ATGs, has a number of non-autophagic functions [125] and that the effects of conditional deletion of Atg7 in dopamine neurons therefore may not (only) be related to effects on autophagy. In conclusion, more studies are needed to highlight the complex role of autophagy in neurodegeneration.

#### 2.2.2. Metabolic Syndrome Diseases

Metabolic syndrome is the name for a cluster of multifactorial diseases characterized by several risk factors (high blood pressure, high triglycerides, low levels of high-density lipoprotein cholesterol, high blood sugar and /or visceral distribution of body fat) [126]. This condition increases the risk of heart disease, obesity, and type 2 diabetes. There is an increased number of studies showing that autophagy is important for the maintenance of metabolism and that it has a protective role on metabolically active organs such as the pancreas, adipose tissue, skeletal muscle, and liver. Therefore, it is unsurprising that systemic enhanced autophagy is beneficial for the organism and improves the life span of aged mice [127,128]. It has been proposed to treat metabolic syndrome diseases by activating autophagy via caloric restriction, intermittent fasting, physical exercise and pharmacological means [40]. Indeed, recently, Lim et al. demonstrated that enhancing autophagy in metabolic syndrome diseases and in an obesity mouse model improves the metabolic profile [129].

#### 2.2.3. Autoimmune Diseases

Autoimmune diseases are characterized by the abnormal recognition of self-components as foreign, inducing immune antigen-driven response against healthy cells and tissues [130]. These diseases are chronic and present with a wide range of symptoms. One of the challenges in treating autoimmune diseases is to find potent immunomodulators that do not alter the integrity of the immune cells. Unfortunately, most of the effective drugs are immunosuppressants that shut down the entire immune system (reviewed in [39]). Interestingly, several studies demonstrated that autophagy (macroautophagy and CMA) are important for the differentiation and activation of lymphocytes [39,111,112,113,114,115,116,117,118]. In accordance, autophagy dysregulation has been described in various autoimmune diseases and some of the current treatment suggestions include autophagy regulators [39]. The peptide P140/Lupuzor that directly acts on CMA [131,132], significantly improved the clinical response in patient with systemic lupus erythematosus diseas [133].

#### 2.2.4. Mitochondrial Diseases

Mitochondrial diseases are rare conditions caused by dysfunction of the oxidative phosphorylation system. In cellular models of these pathologies, trans-mitochondrial cybrids and human skin-derived fibroblasts showed evidence of altered autophagic flux, accumulation of autophagy markers, and higher mitophagic activity [134,135,136,137,138,139]. In addition, deregulated autophagy has been described in animal models of mitochondrial diseases: (i) an autophagic starvation-like response and mitophagy in the skeletal muscle of a mouse model of mitochondrial myopathy [140,141], and (ii) increased autophagosome numbers in retinal ganglion cells of a murine model of dominant optic atrophy [142]. These observations suggest that altered autophagy might play a role in the pathophysiology of mitochondrial diseases.

## 3. Current Autophagy Markers and Their Limitations

Various methods and markers have been described and extensive guidelines have been produced to guide researchers in the assessment of autophagic activity in different experimental models [143,144]. In this review, we will focus on methods employed to measure autophagy in primary patient samples and in the pre-clinical setting.

*Transmission Electron microscopy* (TEM) is a gold standard method to demonstrate autophagosome formation. TEM reveals the morphology of autophagic structures in the cells and their positioning relative to other cellular components. Autophagosomes appear as double- or multi-membrane vesicles. It is currently unclear whether TEM is a suitable technique to determine autophagy in patient tissue. The sample preparation can lead to artifacts and the double membrane is harder to detect in formalin-fixed samples. Therefore, the operator needs to be well-trained in using TEM and also in the analysis and interpretation of the images [145]. A recent study has reported that snap-frozen cryo-fixation of tissue pieces followed by 1.5% paraformaldehyde post-fixation can help to reveal autophagosome structures [146]. TEM is however so labor-intensive that it is unlikely to become a routine clinical praxis for determining autophagy in patient samples.

*Immunohistochemistry* (IHC) and *Immunofluorescence microscopy* (IF) are routinely used in formalin-fixed paraffin-embedded (FFPE) tissue. FFPE samples are an extensive resource in most hospitals. Therefore, there is a lot of interest in using this type of samples for pre-clinical studies. The ATG proteins MAP1LC3B, p62/SQSMT1, Beclin-1 and LAMP2A are frequently stained and used to assess the status of autophagy [52,147,148,149,150,151,152,153]. These proteins are unfortunately not specific to autophagy and therefore interpretation of autophagic activity based on ATG protein levels is still under discussion. For instance, several studies demonstrated that Beclin-1 is a potent biomarker in several cancers [154,155,156,157,158,159,160]. Unfortunately, while Beclin-1 is an important component of the class III PI3K complex, it has also been shown to bind to BCL-2 inhibiting its autophagic function [161,162]. Furthermore, Beclin-1 and ATG5 can be cleaved and thereby acquire pro-apoptotic functions [28,29,163]. Indeed, several components of the autophagic machinery, including some ubiquitin-like ATGs, have important non-autophagic cellular functions, implicated in cellular reprogramming, cell survival, and death, modulation of cellular traffic, protein secretion, cell signaling, transcription, translation and membrane reorganization [164]. Importantly, using a snapshot of ATG gene expression to quantify a dynamic process clearly has its limitations. For instance, high MAP1LC3B levels could result from either an increase or an impairment in the autophagy flux [165,166]. Indeed, accumulation of an autophagy component that is not degraded during the process (e.g., ATG5, ATG12, Beclin-1) may reflect either a block in autophagy or a high turnover. Moreover, antibodies tested to specifically detect ATG8 family members in immunoblotting assays need to be re-evaluated in formalin-fixed paraffin-embedded tissue. It is therefore difficult to interpret and draw conclusions about autophagy on the sole basis of the abundance of ATG proteins in primary human samples.

*Western blotting* (WB) is frequently used in autophagy assessments. Indeed, it is possible to detect and distinguish the non-lipidated and the lipidated, autophagosome-associated form of ATG8 family members by WB due to the differential migration rate of the two forms. As previously mentioned, the specificity of the antibody is key to obtaining conclusive results [167]. Furthermore, WB requires a substantial amount of cellular material, which may be problematic to obtain from primary patient samples. Moreover, the results will reflect an average from the cells that are in the sample, and thus potential differences across different cell types in the patient sample cannot be detected and potentially important differences in a subset of the sample may be masked. It is possible to detect ATG8 by WB of proteins extracted from FFPE tissue although it is not an easy task with potential protein fragmentation and low protein yields [168]. In addition, altered ATG8 levels on a WB may be the result of altered transcription or translation of the protein instead of altered autophagy. Finally, an increase in autophagosome formation does not necessarily mean an increase in degradation of its content.

*Flow cytometry* allows the recording of high content and multiparametric sample detections and is extensively used for non-adherent cells. Membrane-associated MAP1LC3B-II can be measured after extraction of non-membrane-bound ATG8 using a detergent such as saponin for instance as demonstrated in EGFP-MAP1LC3B reporter expressing cells [169]. While this high-throughput method can be easily used in cells growing in suspension or liquid biopsies, measuring autophagy activity by flow cytometry in adherent cells or FFPE tissue is challenging. Furthermore, microdissection of FFPE tissue to prepare them for flow cytometry can be a tremendous task, but still represents an alternative [170].

To summarize, all the above-mentioned methods have their limitations in assessing autophagic activity in tissue. Measurement of MAP1LC3B and MAP1LC3B-II levels is frequently used to assess autophagy, but this is insufficient. Of note, focus in pre-clinical and clinical studies has so far mostly been on assessing canonical macroautophagy, while an increasing amount of data point to important roles of selective macroautophagy or other types of autophagy, including CMA. Unfortunately, there are no robust markers to measure autophagy activity in archived tissue and *in vivo*. Researchers must rely on their knowledge and expertise, interpret their data with caution, and integrate their interpretation with functional and clinical data.

In addition to the difficulty in measuring autophagy in primary samples, ATG genes also function in membrane biology (reviewed in [125,164,171,172]), such as secretion and endocytosis and have also been associated with cell death mechanism and DNA repair responses. Therefore, non-autophagy functions must be considered besides alterations of autophagic activity when autophagy-related genes are deregulated.

We will discuss some ideas to overcome this difficult task in the next section.

## 4. New Avenues for Better Autophagy Markers

Scientists in translational biomedical research often seek better therapeutic targets by comparing cell signaling pathways in healthy and diseased tissue. In recent years, autophagy modulation has turned out to be an attractive option to tackle a large number of diseases. Accurately monitoring autophagic activity in tissues will likely implement new treatment strategies and a better understanding of the role of autophagy in a given disease. Therefore, reliable autophagy markers, improved staining protocols, and agreed interpretation for primary human samples are urgently needed before we can really determine the contribution of autophagy to a specific disease or treatment. New autophagy markers will also be of great value for in vitro and in vivo experimentation/research.

As stated earlier, most of the published pre-clinical and clinical studies rely on MAP1LC3B-dependent methods to assess autophagy. While for some types of autophagy and experimental setting this marker is valuable, our current knowledge on autophagy suggests that MAP1LC3B does not always reflect the actual autophagic flux. Therefore, we propose to increase the number and diversity of markers in order to not only improve autophagic flux measurement but also to assess different types of autophagy that contribute to a given phenotype. We should, for example, include additional ATG8 family members, other ATGs and autophagic substrates and CMA markers such as LAMP2A and HSC70 (HSPA8).

All autophagy subtypes are multistep processes, and it is therefore reasonable to include ATG proteins operating in different phases. Furthermore, lysosomes are central for the degradation efficiency via autophagy. Lysosomal markers should be used together with autophagy markers to get a better indication as to whether the autophagy machinery is fully functional. This includes assessing lysosomal integrity in the tested samples [173,174,175,176]. In addition, a critical step for macroautophagy is the fusion of the autophagosome with the lysosome. Therefore, techniques to detect pH in the autophagic vesicles may be useful to detect the fusion of the autophagosome (neutral pH) to the lysosome (low pH) [177].

Other key players in the process are autophagy cargo receptors. While the majority of studies rely on p62/SQSTM1, one should not forget additional receptors such as NBR1, NDP52, Optineurin [178] and NCOA4 [179], and more recently identified receptors should be added to this list, e.g., ER-phagy receptors. Knowing the specific cargo receptor involved in the autophagy pathway will point to the specific substrates degraded in the experimental setting or in a particular malignancy. Indeed, monitoring the expression levels of autophagy targets may provide information on autophagic flux when combined with analyzing proteins of the autophagy machinery. The notion of autophagic flux includes a time dimension and the autophagosomal pool size per cell and the current techniques may fail in measuring autophagy in a snapshot [165]. Therefore, the quantification of proteins that are degraded during the process (i.e. amount of substrate will inversely correlate to the degradation activity of autophagy) coupled with the quantification of autophagosomes using marker proteins that are maintained during autophagy (mediators of autophagy amounts correlate with autophagosome amount) will help to gain knowledge on the autophagic activity. For instance, detection of low concentrations of autophagy substrates (e.g., p62/SQSTM1) together with the presence of high amounts of non-substrate autophagy proteins (e.g., ATG5) may indicate high autophagy activity. Importantly, hypotheses based on any particular protein ratio should be supported by experimental systems in which mechanistic relationships can be tested in the relevant cell type. In general, it is most promising to investigate substrate proteins that are (1) only inefficiently degraded by breakdown mechanisms other than autophagy, (2) produced at stable rates, and (3) able to accumulate within a cell without undergoing secretion or rapidly causing cell death. Potentially useful candidates of this type of autophagy substrates are components of large protein complexes such as the subunits of the CCT/TRiC chaperonin and proteasomes [180,181].

Given the limitations of antibody-based methods, mass-spectrometric (MS) analysis of FFPE tissues may offer important advantages due to a higher specificity and broader scope, that is, the capacity to detect many proteins in parallel. FFPE tissue MS protocols have been reported [182,183] and their application in the assessment of autophagy should be tested in the near future.

In some contexts, autophagy affects gene expression and therefore it is appropriate to measure the mRNA expression of autophagy-regulated genes. Transcription factors targeted by autophagy provide the opportunity to couple protein detection methods with RNA-seq of their target genes. For instance, HIF-1 alpha is a known CMA target and GATA4 is a senescence regulator that is degraded by selective macroautophagy under basal conditions in human fibroblasts [184]. Therefore, analysis of the mRNA levels of target genes and protein expression of the transcription factor in question, combined with analyses of autophagy-related genes, will help to delineate the autophagy activity involved in specific diseases. Apart from correlating expression of certain genes with autophagic activity, the quantification of mRNAs allows one to estimate the synthesis of autophagy substrates. This helps to evaluate whether high abundance of a substrate, e.g., p62/SQSTM1, is caused by inefficient autophagic degradation or high synthesis (or both).

Many ATG genes are modified at the posttranslational level in a way that influences autophagic activity (Reviewed in [185,186]). Therefore, the use of antibodies recognizing specific posttranslational modifications in ATG proteins will provide a better picture of the autophagic activity in tissues and patient samples. It is desirable to develop antibodies that are specific for lipidated MAP1LC3 and GABARAPs (LC3-II/GABARAPs-II as opposed to LC3-I/GABARAPs-I), protein conjugates such as ATG12–ATG5 (as opposed to free ATG12 and ATG5), and other posttranslational modifications found in ATG proteins (e.g., phosphorylation, acetylation, O-GlcNAcylation etc.).

*In-situ* hybridization (ISH) identifies mRNA expression in tissue samples and can be used to assign gene expression to specific regions of an organ. Furthermore, ISH can be used if specific antibodies are not available. For instance, *Alfy* expression levels in mouse brain during development were detected by ISH [187]. Therefore, ISH can be used as a method complementary to IHC. Advanced methods such as RNAScope ISH offer high sensitivity at relatively low technical complexity.

Finally, it is not only the expression levels of proteins that is important but also their intracellular localization, which notably can affect the function of ATG proteins. Therefore, whenever possible the cellular compartmentation of the proteins of interest should be monitored.

## 5. The Pathologists’ Corner

**Figure 2:**
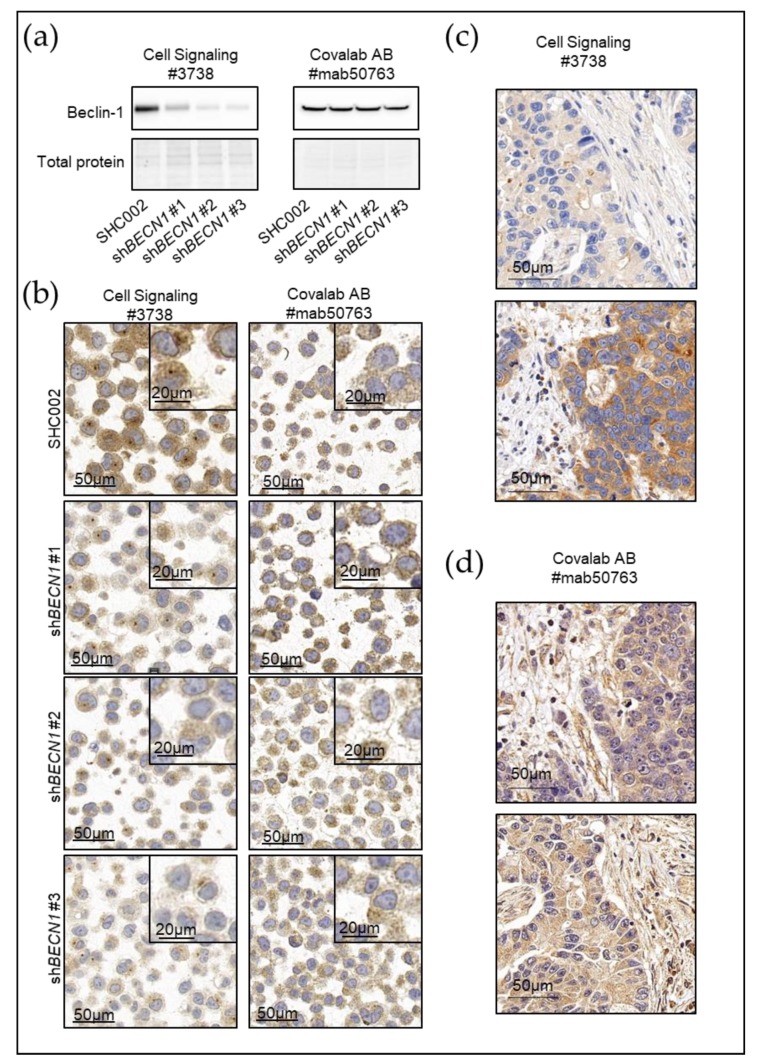
Specificity test of two different anti-Beclin-1 antibodies in FLO-1 human oesophageal adenocarcinoma cells and in primary gastric cancer. FLO-1 cells were transduced with a non-targeting, scrambled shRNA (SHC002) or 3 independent shRNAs targeting *BECN1*. (**a**) Total lysates were subjected to immunoblotting using anti-Beclin-1 from Cell Signaling Technologies (left panel) or Covalab (right panel). Stain-Free Total protein is shown as a loading control, (**b**) cell pellets were fixed and subjected to immunohistochemistry using anti-Beclin-1 from Cell Signaling Technologies (left panel) or Covalab (right panel). (**c**,**d**) 2 Primary Gastric Cancer samples were fixed and subjected to immunohistochemistry using anti-Beclin-1 from Cell Signaling Technologies (**c**) or Covalab (**d**). The results indicate that the Cell Signaling Technologies antibody specifically recognizes Beclin-1 in both Western Blotting (WB) and immunohistochemistry (IHC), whereas the Covalab antibody shows no or very poor specificity with either method. Furthermore, scoring results of primary Gastric Tumors gave different results from one case low and on high with Cell Signaling Technologies antibody to both in a moderate range with the Covalab antibody. The Cell Signaling Technologies antibody would therefore be the more reliable antibody to use in assessing Beclin-1 levels in human oesophageal adenocarcinoma patient samples. The experiments were performed by Félice Janser and José Galván in the lab of Rupert Langer. Representative pictures are shown.

Clinical pathologists and researchers should combine their efforts in order to better understand the autophagy machinery and its impact in human health. Basic scientists depend on the expertise of pathologists in assessing cell and tissue morphology and identifying alterations associated with disease. Improved collaborations between clinicians and researchers will facilitate the generation of clinically relevant hypotheses in the autophagy field. Furthermore, trained pathologists can support the autophagy researchers in developing standardized staining and scoring protocols allowing for better reproducibility and comparability of basic research and clinical studies. Autophagy cannot be detected in routine H&E-stained FFPE tissues and therefore relies on biomarkers detection by IHC [188]. Reliable antibody testing should be done to validate the specificity and the sensitivity of antibodies used in different experimental set-ups and in tissue staining. For instance, we have observed that anti-Beclin-1 antibodies may give very different results and the specificity of the antibodies should be tested by both western blotting (Figure 2a) and IHC of cell lines (Figure 2b) and more importantly IHC of primary samples (Figure 2c,d). Indeed, while Cell Signaling Technologies antibody (#3738) detects knockdown in FLOT-1 cell line by western blot and IHC, the Covalab antibody (#mab50763) does not. In concordance, IHC of two gastric tumors demonstrated a case with low and high Beclin-1 expression with the Cell Signaling Technologies antibody (Figure 2c) while no significant difference was detected with the Covalab antibody (Figure 2d). Whenever possible, the specificity of antibodies for a particular protein should be validated with respective knockdown/knockout cell models and/or by ectopic expression of the protein before patient cohorts are screened (Figure 2).

Working hand-in-hand will undoubtedly increase the number of autophagy markers that are reliable and relevant for a given disease or experimental sample.

## 6. Gathering New Knowledge and Tools for a Better Autophagy Assessment

A broad use of autophagy markers by diagnostic laboratories and pharma companies should be highly reliable, easy to handle and cost-effective. The first step to design the best strategy to assess autophagy is to identify the autophagy pathway involved in a particular model, if possible, with a pre-test *in vitro*, for example, by identifying the essential ATG genes.

In-depth understanding of the autophagy pathway at the molecular level will help to design specific markers. Indeed, understanding the core autophagy components and the upstream and downstream regulators will provide new tools to dissect the autophagy response in a given disease context.

Gene panels are increasingly being developed for cancer diagnosis, prognostics, and to guide treatment. For example, in breast cancer several multigene prognostic tests have been developed to improve stratification of patients and treatment decisions (reviewed in [189]). Since autophagy cannot be deduced by the use of single markers in archived tissue, it will be very interesting for autophagy researchers to identify gene “signatures” that can predict autophagic activity and even response to autophagy-targeted treatment.

Furthermore, designing autophagy detection kits for non-invasive samples such as plasma or liquid biopsies instead of tissue markers will facilitate less invasive sampling. Interestingly, in patients with multiple sclerosis, the levels of ATG5 and Parkin in the serum and the cerebrospinal fluid were found to be significantly higher than in the control cohorts [190,191]. In addition serum levels of Beclin-1 are related to the stage of diabetic kidney disease and inversely correlate with a kidney dysfunction marker albuminuria in these patients [192].

In conclusion, a better overall understanding of autophagic activity in patients and patient tissue may open up new diagnostic, prognostic and therapeutic strategies.

## 7. Improving Autophagy Assessment by Sharing Knowledge

The field of autophagy is rapidly growing and many groups have done tremendous work to link autophagy to disease. Therefore, a platform that summarize all the current knowledge is urgently needed. An autophagy atlas that will give researchers the opportunity to implement current knowledge in their experimental design and autophagy quantification would be a first step in the right direction. Furthermore, this platform will improve collaborations between autophagy groups working on similar diseases and therefore increase the impact of their research.

## 8. Conclusions

While autophagy is still a growing field, we should not neglect the need to improve currently available autophagy markers as well as to identify novel ones for tissue samples. A team effort is needed to provide improved autophagy assays to better correlate autophagy activity with disease outcome and therapy response in translational biomedical research. Furthermore, guidelines to how to proceed with pre-clinical and clinical samples and to interpret the data will be essential.

## Figures and Tables

**Table 1:** Summary of the effect of autophagy on cancer discussed in the present review. For more detailed information, please see the indicated list of recent review articles [38,39,40,41,42,43,44,45,46,47].

Type of Autophagy	Level of Activity	Type of Tumor	Role of Autophagy
Macroautophagy	Low	Solid Tumors	Beclin-1 deficiency in mice increase tumor incidence [48]
			Low Beclin-1 expression levels in human brain tumors [65]
			Low Beclin1 mRNA levels iscorrelated with a poor prognosis in HER2-enriched breast tumors [66]
			Low macroautophagy activity in HSCs increases risk of developing hematopoietic malignancies [86,87,88,89,90,91]
		Leukemia and Lymphomas	Low ATG gene expression in AML [90,94,95,96,97]
			Accelerated leukemia development in mouse model with impaired macroautophagy [96,97,98,99]
			ATG gene mutations found in AML [97,100,101]
	High	Solid Tumors	Therapy-induced macroautophagy enhances tumor cell survival [49,50,73,74,94,95]
			RAS-driven tumors are autophagy-dependent [66,70,71,72]
		Leukemia and Lymphomas	High macroautophagy activity in FLT3-ITD mutant AML patients [102]
			Important for the differentiation and activation of lymphocytes [39,111,112,113,114,115,116,117,118]
CMA	Low	Solid Tumors	Lamp2a deficiency in a mouse model increase liver tumor incidence [63]
	High	Solid Tumors	High LAMP2A expression commonly found in cancers [52]
			Therapy-induced CMA promote tumor cell survival [56]
			CMA activity contributes to tumor cell proliferation and metastatic potential [52,60,61,62]
